# Supplementary material for: Tracking the Evolution of Cutaneous Melanoma by Multiparameter Flow Sorting and Genomic Profiling
Source: Int J Mol Sci. 2025 Feb 19;26(4):1758. doi: 10.3390/ijms26041758 (PMC11855598; doi:10.3390/ijms26041758)
Supplement: Supplementary file 1 [file ijms-26-01758-s001.zip › Supplementary Figure S2.pdf]

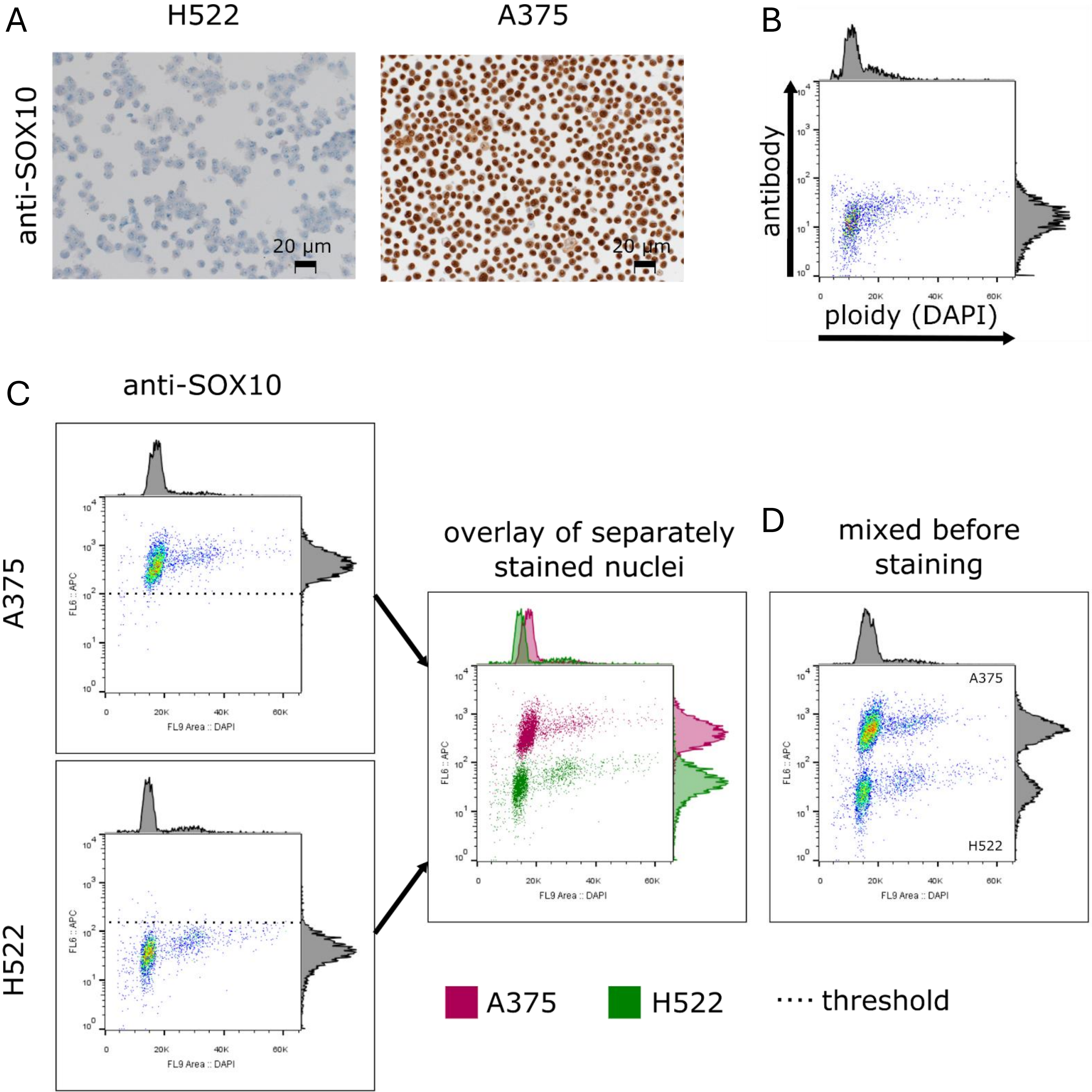
A
H522
A375
anti-SOX10
20 µm
20 µm
B
antibody
ploidy (DAPI)
C
anti-SOX10
A375
H522
FL6 :: APC
FL9 Area :: DAPI
overlay of separately stained nuclei
D
mixed before staining
A375
H522
A375
H522
threshold
