# Supplementary material for: Tracking the Evolution of Cutaneous Melanoma by Multiparameter Flow Sorting and Genomic Profiling
Source: Int J Mol Sci. 2025 Feb 19;26(4):1758. doi: 10.3390/ijms26041758 (PMC11855598; doi:10.3390/ijms26041758)
Supplement: Supplementary file 1 [file ijms-26-01758-s001.zip › Supplementary_information.pdf]

Supplementary Figure S1. Sorted tumor populations with SOX10 and DAPI of A) Patient 2 and B) Patient 3. PST = primary site tumor, MT = metastasis tumor. C) Plot showing the levels of tumor contamination in normal samples using DeTiN software for Patient 1(left), Patient 2 (middle), and Patient 3 right (right).

Supplementary Figure S2. A-D) Multiparameter nuclei flow cytometry with anti-SOX10 antibody in cell lines A375 and H522. (A) IHC for SOX10 on cytospins from the cell lines H522 (SOX10-negative) and A375 (SOX10-positive), respectively. (B,C) Multiparameter flow cytometry of nuclei extracted from both cell lines and stained for DNA content with DAPI (x-axis) and isotype control (b, y-axis) or anti-SOX10 antibody (c, y-axis). (D) The A375 SOX-10 positive nuclei are separated from the SOX-10 negative H522 nuclei by FACS with the anti-SOX10 antibody.

Supplementary Figure S3. Venn diagrams showing the numbers of synonymous and non-synonymous mutations detected using WES in all three patients.

Supplementary Figure S4. Phylogenetic trees calculated based on the CCF of synonymous and non-synonymous mutations for Patients 2 and 3.
