# Supplementary figures and images for: Tracking the Evolution of Cutaneous Melanoma by Multiparameter Flow Sorting and Genomic Profiling
Source: Int J Mol Sci. 2025 Feb 19;26(4):1758. doi: 10.3390/ijms26041758 (PMC11855598; doi:10.3390/ijms26041758)

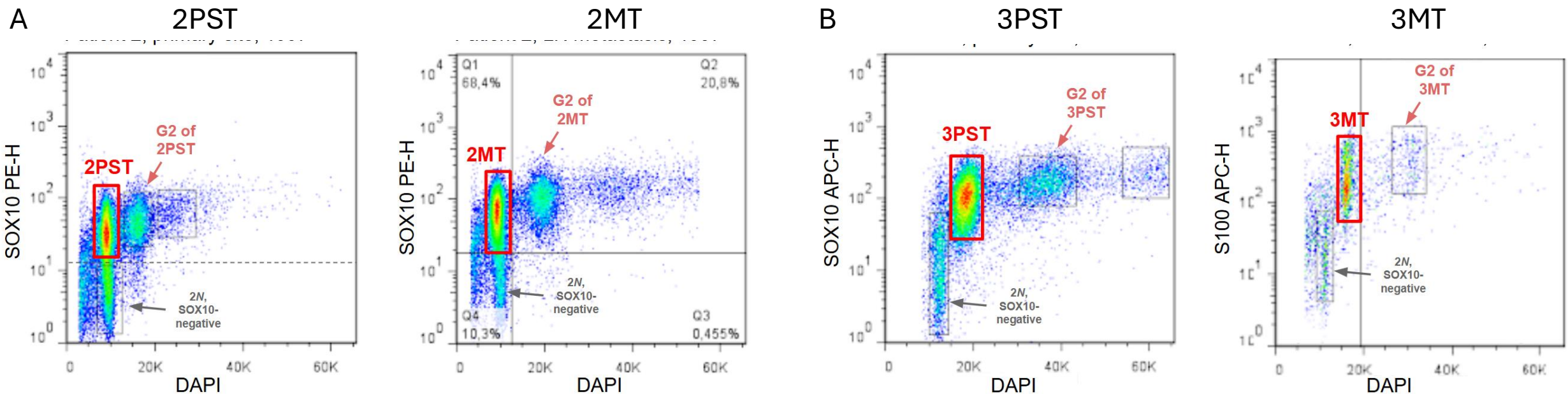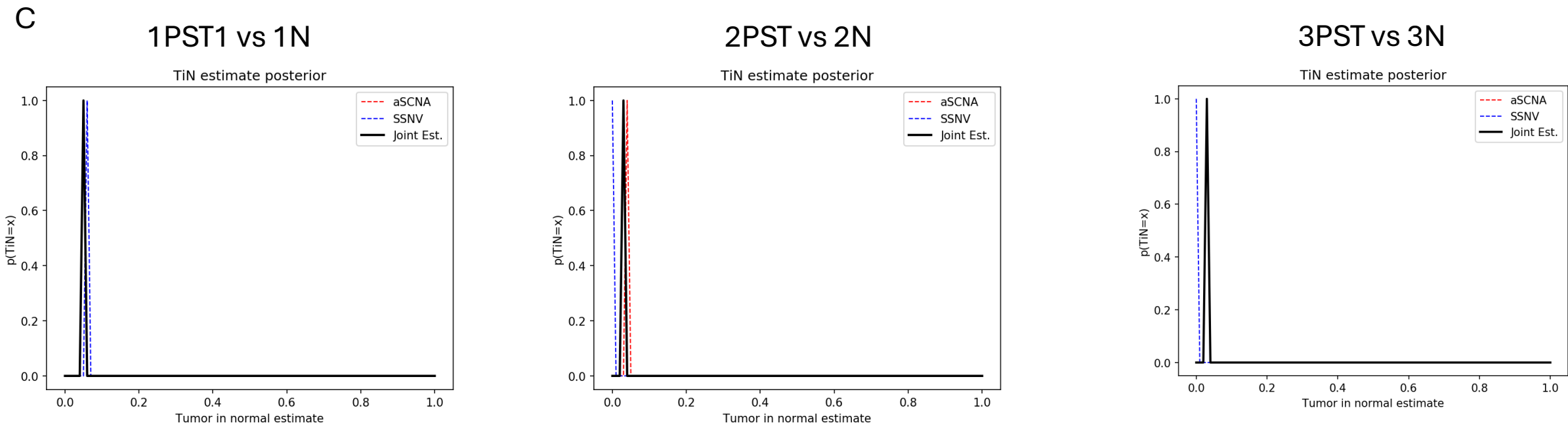

Supplement: Supplementary file 1 [file ijms-26-01758-s001.zip › Supplementary Figure S1.pdf]

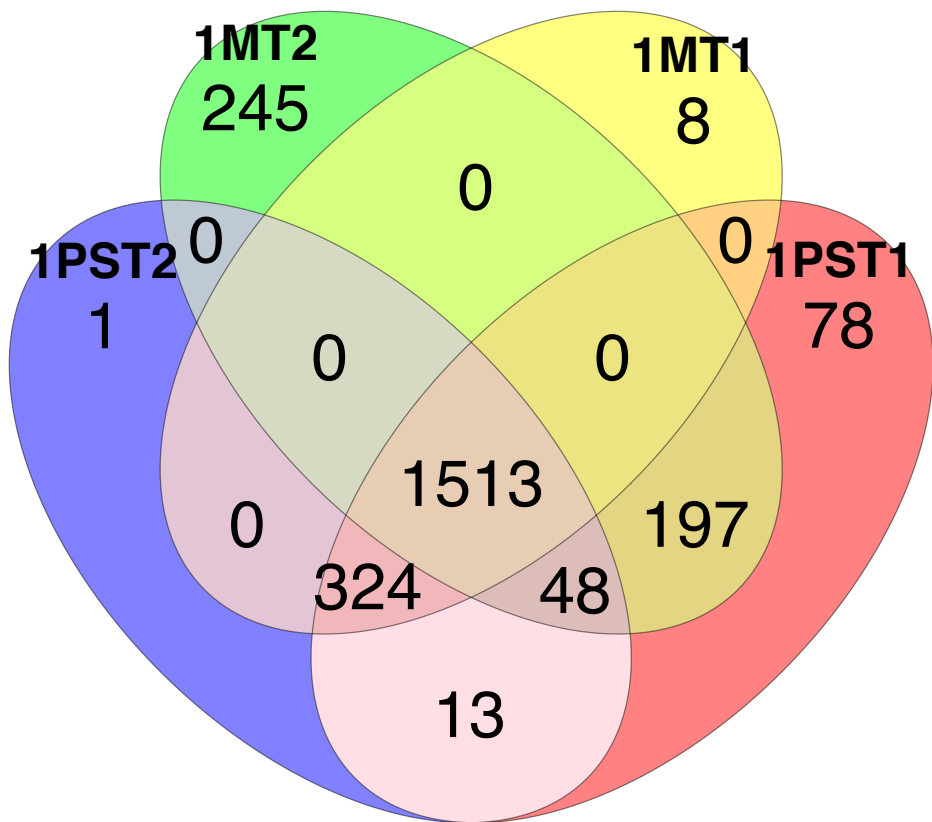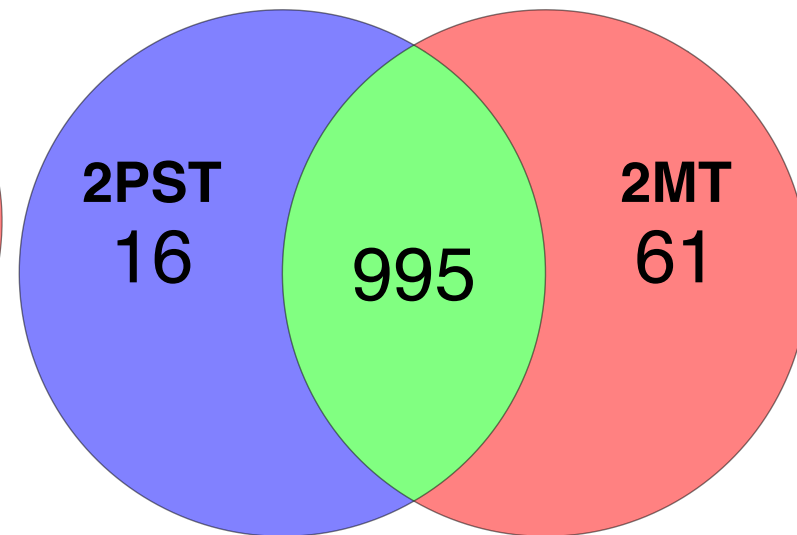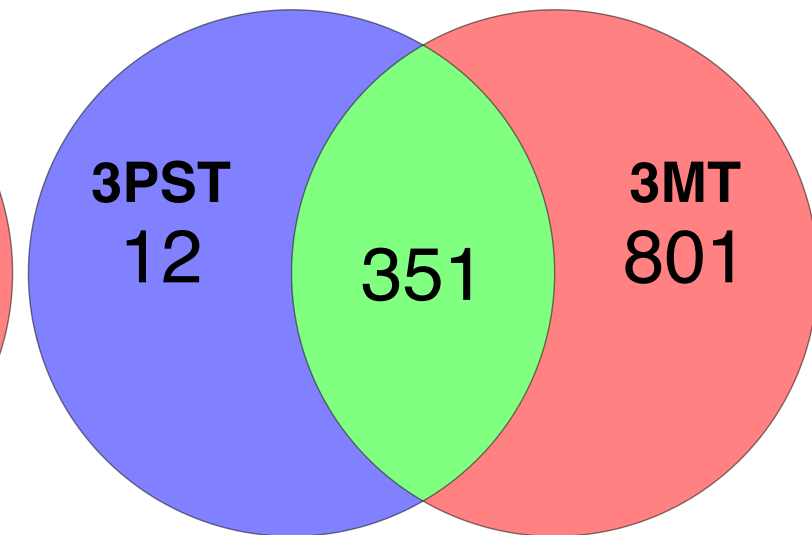

Supplement: Supplementary file 1 [file ijms-26-01758-s001.zip › Supplementary Figure S3.pdf]

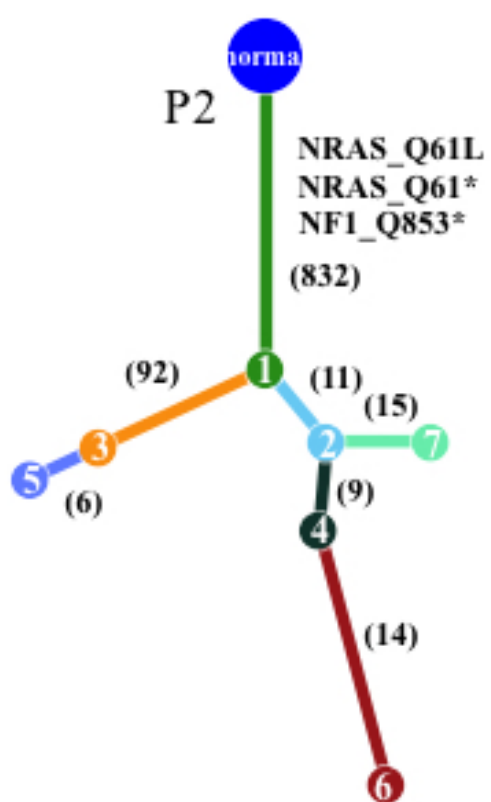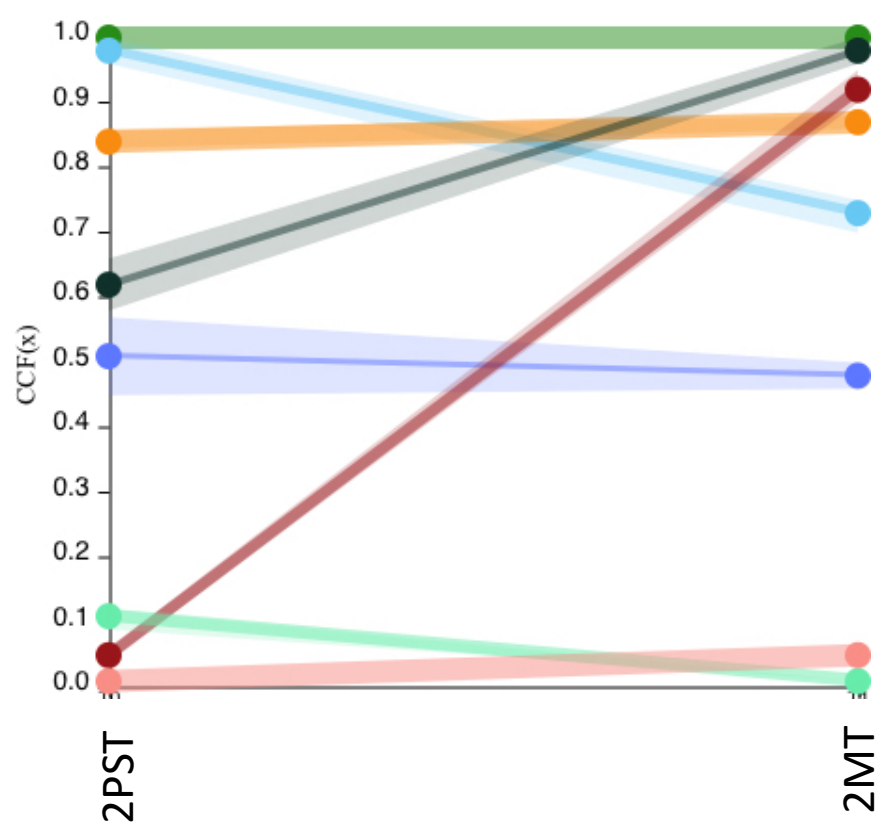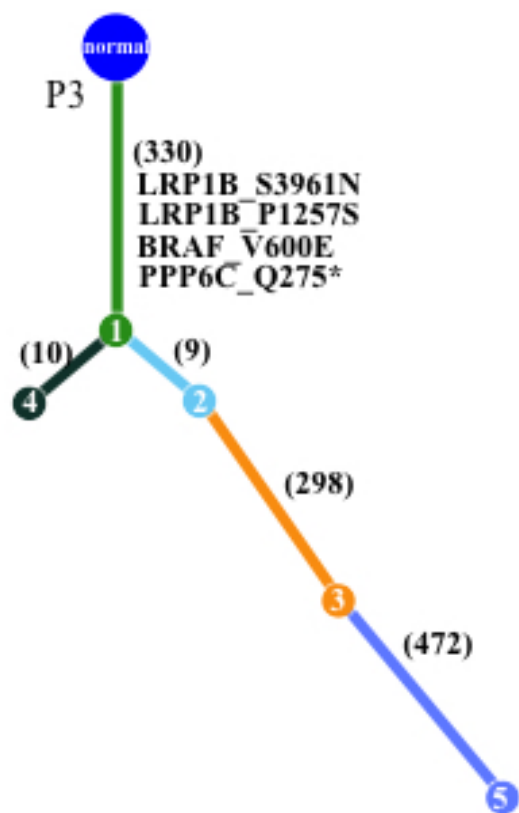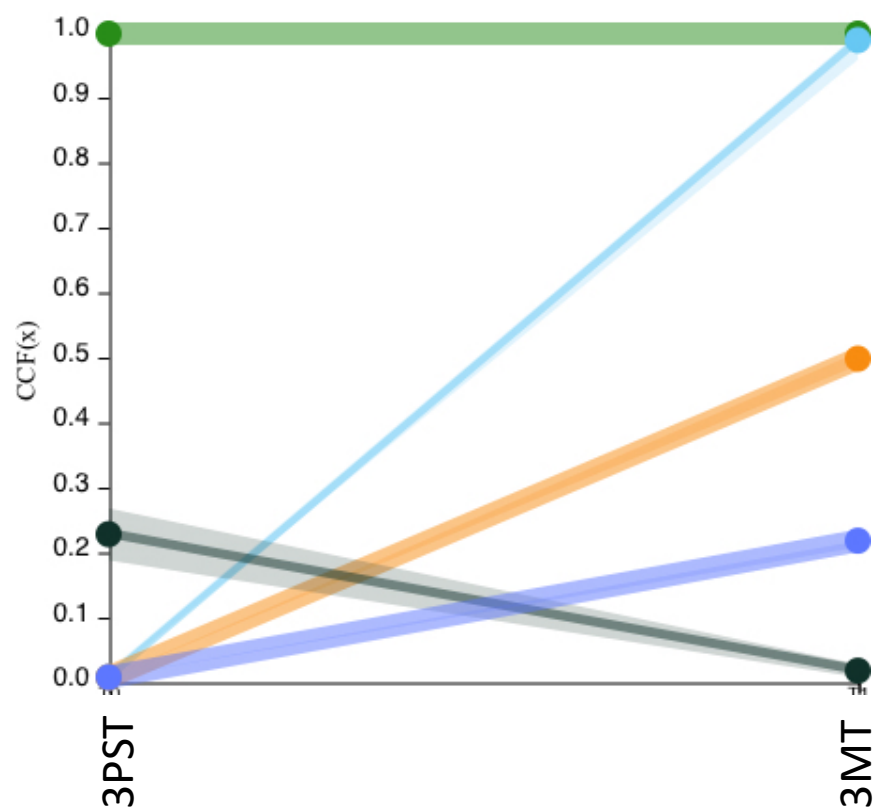

Supplement: Supplementary file 1 [file ijms-26-01758-s001.zip › Supplementary Figure S4.pdf]
